# Efficacy and safety of antibody-drug conjugates for HER2-expressing advanced gastric and gastroesophageal junction adenocarcinoma: a systematic review and meta-analysis

**DOI:** 10.3389/fphar.2025.1668511

**Published:** 2025-09-17

**Authors:** Danxue Huang, Feilong Sun, Su Li, Liyuan Ke

**Affiliations:** ^1^ Department of Pharmacy, Cancer Hospital of China Medical University, Liaoning Cancer Hospital and Institute, Shenyang, China; ^2^ Jiangsu Hengrui Pharmaceuticals Co., LTD, Lianyungang, China

**Keywords:** gastric cancer, antibody-drug conjugates, gastroesophageal junction adenocarcinoma, meta-analysis, HER2-expressing

## Abstract

**Background:**

Antibody-drug conjugates (ADCs) represent a promising therapeutic modality for gastric cancer. Given the highly heterogeneous nature of this malignancy, the efficacy and safety profile of ADC treatment warrant comprehensive evaluation.

**Methods:**

A systematic search of online databases identified prospective trials published through June 2025. Pooled estimates for OS, PFS, ORR, DCR, and TRAEs were derived using a random-effects model. Subgroup analyses were performed, stratified according to HER2 status, primary tumor location, line of therapy, and use of combination treatment.

**Results:**

A total of 1779 patients from 13 prospective trials (18 reports) were included. The pooled ORR was 67% (95% CI: 53%–82%) for first-line ADC therapy, 40% (95% CI: 29%–51%) for second-line regimens, and 27% (95% CI: 16%–38%) for third-line regimens. In second-line or later therapy, HER2-positive patients achieved a superior ORR relative to HER2-low subgroups (39%, 30%–47% vs. 25%, 11%–39%). The overall pooled median OS was 11.95 months (95% CI: 9.93-13.96), with a median PFS of 4.94 months (95% CI: 3.92-5.96). Stratification by line of therapy revealed a median OS of 19.67 months (95% CI: 15.79-23.55) for first-line *versus* 11.65 months (8.09-15.22) for second-line and 9.37 months (7.38-11.37) for third-line, with corresponding median PFS of 10.57 months (6.37-14.77) vs. 4.13 months (2.43-5.83) and 4.50 months (3.51-5.50) respectively. TRAEs occurred in 98% (95% CI: 96%–100%) of patients (any-grade), with grade 3–5 events in 60% (52%–69%).

**Conclusion:**

This meta-analysis establishes ADCs as a promising therapeutic approach for advanced gastric or gastroesophageal junction cancer (GC/GEJC), demonstrating efficacy in both HER2-positive and HER2-low patient populations.

**Systematic review registration:**

https://www.crd.york.ac.uk/PROSPERO/view/CRD420251066208, identifier CRD420251066208.

## 1 Introduction

Gastric cancer (GC) is the fifth most common cancer and the fourth leading cause of cancer-related death worldwide (Sung et al., 2021; Morgan et al., 2022). Given the subtle nature of its symptoms, most patients with GC are diagnosed at an advanced or metastatic stage (Hundahl et al., 2000). For early-stage GC, therapeutic options include endoscopic mucosal resection (EMR) and endoscopic submucosal dissection (ESD) (Sundar et al., 2025). Advanced cases, however, typically require a combined approach of surgery and chemotherapy to improve 5-year survival. Patients with unresectable GC often receive concurrent radiotherapy and chemotherapy, which has been shown to enhance survival outcomes (López et al., 2023; Guan et al., 2023). HER2 (human epidermal growth factor receptor 2) represents an established therapeutic target in GC management. The large-scale, international HER-EAGLE study revealed a global HER2-positivity rate of 10%–20% in GC, defined as immunohistochemistry (IHC) 3+ or IHC 2+ with positive *in situ* hybridization (ISH) (Kim et al., 2018). HER2-low expression was defined as IHC 2+/ISH-negative or IHC 1+. For locally advanced or metastatic HER2-positive disease, first-line trastuzumab-based regimens represent standard therapy (Bang et al., 2010). Immune checkpoint inhibitors combined with platinum-based doublet chemotherapy have become standard first-line treatment for HER2-negative advanced GC (Janjigian et al., 2021; Rha et al., 2023; Kang et al., 2022; Shitara et al., 2020a). Ramucirumab, administered either as monotherapy or combined with paclitaxel, constitutes an established second-line option (Wilke et al., 2014; Fuchs et al., 2014).

Recent advances in clinical oncology drug development have witnessed the approval of novel antibody-drug conjugates (ADCs) including disitamab vedotin (RC48) (Peng et al., 2021) and trastuzumab deruxtecan (T-DXd) (Shitara et al., 2020b), expanding therapeutic options for advanced-stage disease. ADCs represent a powerful class of cancer drugs. They combine the precision of monoclonal antibodies with potent cytotoxic agents, linked together to deliver the payload directly to tumor cells. A key advantage of many ADCs is their bystander-killing effect. This occurs when the cytotoxic drug escapes the initial target cell and enters neighboring cells, triggering cell death (apoptosis) and helping to overcome challenges posed by tumor heterogeneity. Currently, global clinical trials involve more than 100 ADC candidates; so far, 15 have gained regulatory approval. Significantly, agents like T-DXd, RC48, and IMMU-132 are now options for treating advanced GC (Peng et al., 2021; Shitara et al., 2020b; Hao et al., 2024). Pushing forward with next-generation ADCs and exploring novel treatment approaches is therefore vital. These efforts offer fresh hope for advanced GC patients whose current choices are limited. Recent years have witnessed increasing clinical recognition of HER2-low breast cancer as a distinct therapeutic subtype, stimulating interest in exploring this entity within the GC landscape (Modi et al., 2022; Tara et al., 2020; Yu et al., 2023). Patients with HER2-low GC derive limited benefit from conventional HER2-targeted monoclonal antibodies (Bang et al., 2010), necessitating novel therapeutic strategies. Evidence suggests ADCs may exhibit antitumor activity in this population, potentially mediated through the bystander effect inherent to certain ADC constructs. This phenomenon-whereby cytotoxic payloads released from dying tumor cells exert cytotoxic effects on adjacent cells-proves particularly valuable for eliminating HER2-low cells and addressing tumoral heterogeneity (Li et al., 2016). The phase II C013 trial demonstrated efficacy of RC48 plus toripalimab in pretreated HER2-low gastric/gastroesophageal junction cancer (GC/GEJC) patients (IHC2+/ISH- or IHC1+), reporting an objective response rate (ORR) of 46%, median progression-free survival (PFS) of 5.1 months, and median overall survival (OS) of 14.0 months (Wang et al., 2024).

Contemporary clinical development of ADCs in advanced GC includes ongoing trials evaluating monotherapy and combination regimens. This complex disease exhibits substantial intratumoral genomic and phenotypic heterogeneity, which poses therapeutic challenges. Consequently, clinical outcomes demonstrate significant variability across studies-with some meeting primary endpoints while others report non-significant results. Addressing the unmet need for synthesized evidence, this systematic review and meta-analysis comprehensively assesses ADC efficacy and safety profiles in advanced GC/GEJC. Additionally, we characterize clinical and molecular subpopulations exhibiting differential responses to ADC therapies.

## 2 Materials and methods

This systematic review and meta-analysis was conducted in accordance with PRISMA guidelines (Preferred Reporting Items for Systematic Reviews and Meta-Analyses) and registered prospectively with PROSPERO (International Prospective Register of Systematic Reviews) (CRD420251066208).

### 2.1 Data source and search strategy

Literature searches were conducted in Web of Science, Embase, PubMed, and the Cochrane Library, supplemented by screening abstracts from ESMO (European society of medical oncology) and ASCO (American Society of Clinical Oncology) annual meetings. The search period covered inception through June 2025. The search strategy employed terms related to antibody-drug conjugates (“antibody-drug conjugate”, “ADC”, specific agents like “T-DM1”, “T-DXd”, “trastuzumab deruxtecan”, “disitamab vedotin”, “Trastuzumab emtansine”, “RC-48”, “DS-8201a”, “ARX788”) AND esophagogastric or gastric cancer (“esophagogastric”, “gastric”, “stomach”, “gastro-oesophageal”) AND advanced or unresectable stage (“unresectable”, “advanced”, “metastatic”).

### 2.2 Study selection

Trials were selected if they: 1) were prospective phase I-III studies; 2) enrolled locally advanced/unresectable GC/GEJC patients; 3) HER2 IHC≥1 or ISH positive 4) administered ADCs; 5) reported ≥1 clinical endpoint (OS, PFS, ORR, disease control rate (DCR) or treatment-related adverse events (TRAEs)); and 6) were published in English. Animal studies, non-original research (e.g., reviews, case reports, editorials), and commentaries were excluded.

### 2.3 Data extraction and quality assessment

Two investigators (Huang and Li) independently extracted study characteristics (first author, publication year, design, trial phase, registration number, sample size), patient demographics (region, age, clinical stage), and treatment arm details. Primary outcomes encompassed OS, PFS, ORR, DCR and TRAEs. Methodological quality was appraised using the Cochrane risk-of-bias tool in RevMan 5.4 for randomized trials, with single-arm studies assessed via the modified MINORS criteria.

### 2.4 Statistical analysis

Statistical analyses were performed in Stata 14.0. Pooled estimate with 95% CIs were computed for OS, PFS, ORR, DCR, and TRAEs. The presence of significant heterogeneity was assessed using the Cochran’s Q statistic (with a significance level of *p* < 0.10) and the *I* ^*2*^ statistic. Significant heterogeneity was defined as an *I* ^*2*^ value greater than 50% coupled with a p-value from the Cochran’s Q test of less than 0.10 (Higgins et al., 2003). In such cases, a random-effects model was employed; otherwise, a fixed-effects model was applied. Publication bias was assessed via Begg’s tests (*p* > 0.05 indicating nonsignificance) (Begg and Mazumdar, 1994). To explore potential sources of heterogeneity, univariable meta-regression analyses were performed for the primary outcome (e.g., ORR, OS, PFS) using the following study-level covariates: median age, publication year, and study size. Subgroup analyses stratified combination therapies, line of therapy, primary tumor location, and HER2 status.

## 3 Results

### 3.1 Study selection and characteristics

**FIGURE 1:**
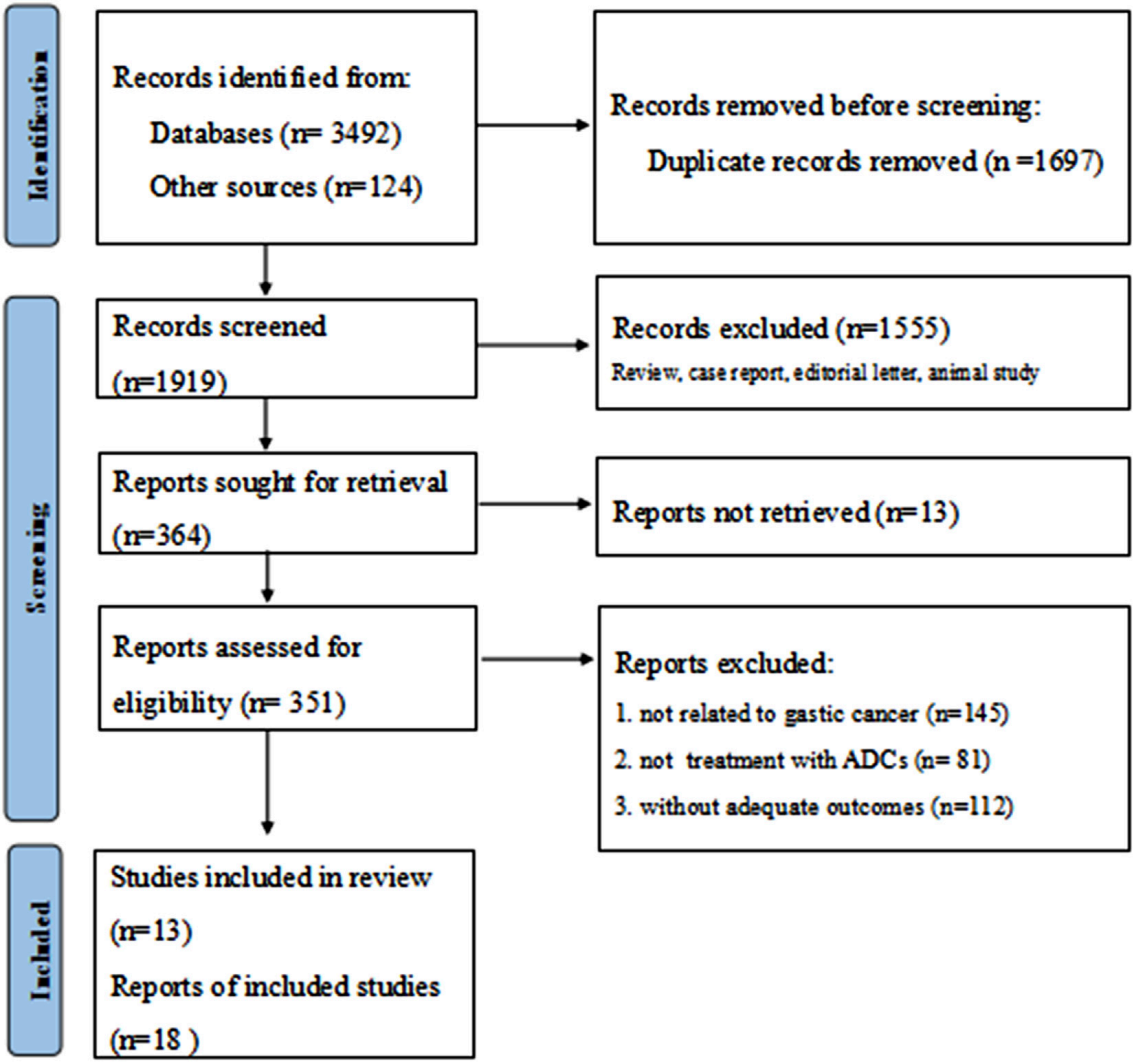
Flow diagram of the screening and selection process.

Following a systematic literature search yielding 3,616 potentially relevant trials, two authors (Huang and Li) independently screened records for eligibility. After excluding irrelevant and duplicate entries, 351 abstracts and articles underwent further assessment. Janjigian et al. (2024) (Janjigian et al., 2024a) reported five cohorts grouped by distinct therapeutic agents, while Yamaguchi et al. (2022) (Yamaguchi et al., 2023) presented two cohorts stratified according to HER2 status. Ultimately, eighteen studies derived from thirteen publications were included in the final analysis (Figure 1) (Peng et al., 2021; Shitara et al., 2020b; Wang et al., 2024; Li et al., 2024; Janjigian et al., 2024a; Thuss-Patience et al., 2017; Van Cutsem et al., 2023; Zhang et al., 2022; Song et al., 2024; Li et al., 2023; Shitara et al., 2025; Yamaguchi et al., 2023; Shen et al., 2023). The included studies comprised four phase I trials, twelve phase II trials, and two phase III trials. These studies collectively enrolled 1779 patients, with a mean age around 60 years. Detailed baseline characteristics are presented in Table 1. All included studies demonstrated high-moderate quality according to MINORS criteria, consistently scoring 14-16 points; specific assessments are provided in Supplementary Table S1; Supplementary Figure S1.

**TABLE 1:** Main characteristic of the eligible studies in the meta-analysis.

Author	Year	Study phase/design	Numbers of parents	Median age	Sex (male vs. Female)	Arm	HER2 status	Median OS (month)	Median PFS (month)	ORR (%)	DCR (%)	NCT number
Li et al. (2024)	2024	II/single-arm	55	65	42 vs. 11	RC48 2.5 mg/kg + Tislelizumab + S-1	IHC3+or IHC2+	NR	NR	90.9%	97.7%	NCT 05586061
Janjigian et al. (2024a)	2024	Ib/II/cohort	43	61	30 vs. 13	T-DXd 6.4 mg/kg	IHC3+or IHC2+/ISH+	18 (10-26)	9 (5-17)	49%	NR	NCT 04379596
Janjigian et al. (2024a)	2024	Ib/II/cohort	42	60	31 vs. 10	T-DXd 6.4 mg/kg +5-FU/Cape	IHC3+or IHC2+/ISH+	23 (16-NE)	20 (10-28)	78%	NR	NCT 04379596
Janjigian et al. (2024a)	2024	Ib/II/cohort	43	65	33 vs. 10	T-DXd 6.4 mg/kg + pembrolizumab+5-FU/Cape	IHC3+or IHC2+/ISH+	16 (9-NE)	8 (4-NE)	58%	NR	NCT 04379596
Janjigian et al. (2024a)	2024	Ib/II/cohort	41	66	33 vs. 8	T-DXd 6.4 mg/kg + pembrolizumab	IHC3+or IHC2+/ISH+	23 (13-NE)	10 (5-18)	63%	NR	NCT 04379596
Janjigian et al. (2024a)	2024	Ib/II/cohort	32	61	29 vs. 3	T-DXd 5.4 mg/kg + pembrolizumab+5-FU/Cape	IHC3+or IHC2+/ISH+	NR	NR	59.4%	NR	NCT 04379596
Thuss-Patience et al. (2017)	2017	II/III/RCT	415	62 vs. 62	272 vs. 73	T-DM1 2.4 mg/kg vs. a taxane	IHC3+or IHC2+/ISH+	7.9 (6.7-9.5) vs. 8.6 (7.1-11.2)	2.7 (1.6-2.7) vs. 2.9 (2.8-4.0)	20.6% vs. 19.6%	NR	NCT 01641939
Van Cutsem et al. (2023)	2024	II/single-arm	79	61	57 vs. 22	T-DXd 6.4 mg/kg	IHC3+or IHC2+/ISH+	12.1 (9.4-15.4)	5.6 (4.2-8.3)	41.8%	81.0%	NCT 04014075
Wang et al. (2024)	2024	I/single-arm	30	60	19 vs. 5	RC48 2.5 mg/kg + toripalimab	HER2 IHC≥1 or ISH +	14.0 (6.3-NE)	5.1 (1.4-7.3)	50%	68%	NCT 04280341
Zhang et al. (2022)	2022	I/single-arm	30	57	22 vs. 8	ARX788 1.3–1.7 mg/kg	IHC3+or IHC2+/ISH+	10.7 (4.8-NE)	4.1 (1.4-6.4)	37.9%	55.2%	CTR20190639
Song et al. (2024)	2023	I/single-arm	13	62	NR	SHR-A1811 1.0–8.0 mg/kg	HER2 IHC≥1 or ISH +	NR	NR	50%	75%	NCT04446260
Li et al. (2023)	2023	I/single-arm	32	60	27 vs. 5	SHR-A1811 6.4 mg/kg	HER2 IHC≥1 or ISH +	NR	NR	43.8%	84.4%	NCT04513223
Shitara et al. (2025)	2025	III/RCT	494	63 vs. 64	187 vs. 59	T-DXd 6.4 mg/kg	IHC3+or IHC2+/ISH+	14.7 (12.1-16.6) vs. 11.0 (9.4-14.2)	6.7 vs. 5.6	44.3% vs. 29.1%	91.9% vs. 75.9%	NCT04704934
Shitara et al. (2020b)	2020	II/RCT	187	65 vs. 66	142 vs. 45	T-DXd 6.4 mg/kg vs. PC	IHC 3+ or IHC 2+/ISH+	12.5 (9.6-14.3) vs. 8.4 (6.9-10.7)	5.6 (4.3-6.9) vs. 3.5 (2.0-4.3)	43% vs. 12%	86% vs. 62%	NCT 03329690
Yamaguchi et al. (2023)	2022	II/cohort	21	64	16 vs. 4	T-DXd 6.4 mg/kg	IHC 3+ or IHC 2+/ISH+	7.8 (4.7-NE)	4.4 (2.7-7.1)	26.3%	89.5%	NCT 03329690
Yamaguchi et al. (2023)	2022	II/cohort	24	59	19 vs. 5	T-DXd 6.4 mg/kg	IHC 2+/ISH– or IHC 1+	8.5 (4.3-10.9)	2.8 (1.5-4.3)	9.5%	71.4%	NCT 03329690
Peng et al. (2021)	2021	II/single-arm	125	58	91 vs. 34	RC48 2.5 mg/kg	IHC 2+/ISH– or IHC 1+	7.9 (6.7-9.9)	4.1 (3.7-4.9)	24.8%	42.4%	NCT 03556345
Shen et al. (2023)	2023	II/single-arm	73	60	55 vs. 18	T-DXd 6.4 mg/kg	IHC 3+ or IHC 2+/ISH+	10.2 (7.2-14.3)	5.7 (4.0-6.8)	28.80%	79.40%	NCT04989816

DCR, disease control rate; HER2, human epidermal growth factor receptor2; IHC, immunohistochemistry; ISH, *in situ* hybridization; NE, not evaluable; NR, no record; ORR, objective response rate; OS, overall survival; PC, physician’s choice; PFS, progression-free survival; RC-48, disitamab vedotin; T-DXd, trastuzumab deruxtecan; T-DM1, trastuzumab emtansine; 5-FU, 5-fluorourcil; Cape, capecitabine.

### 3.2 ORR and DCR

**FIGURE 2:**
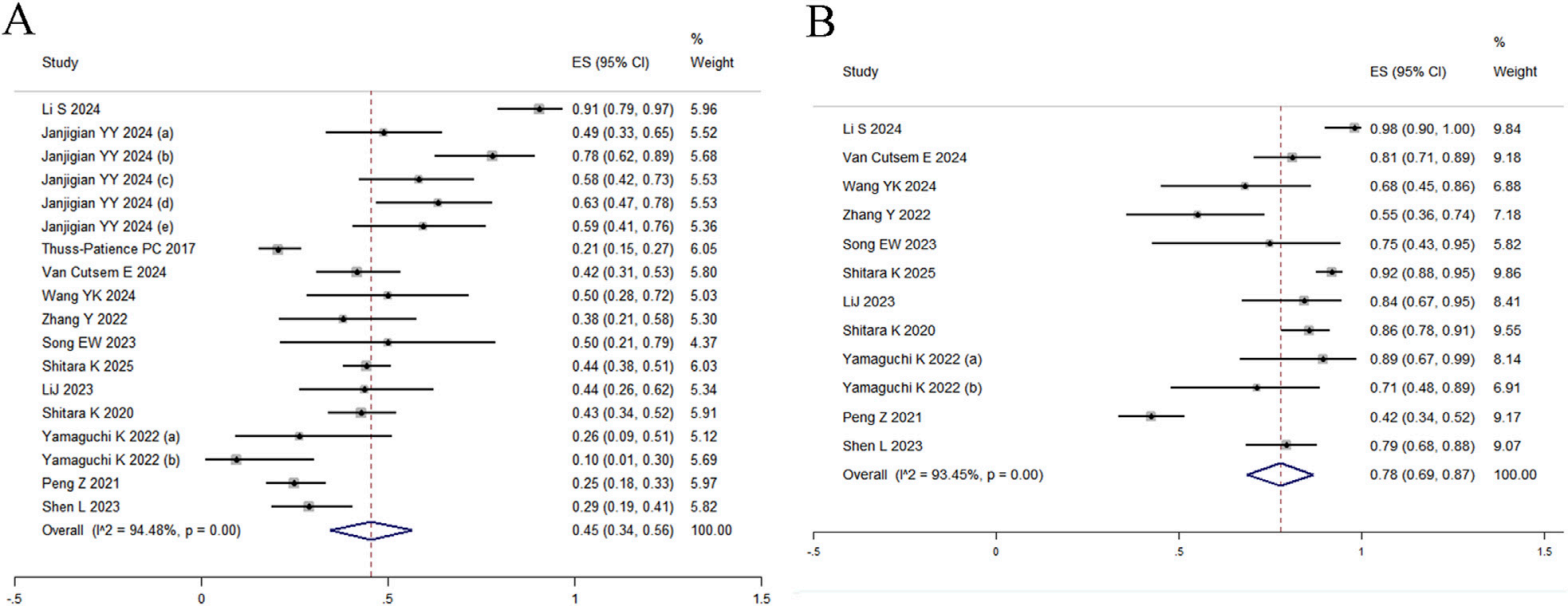
Forest plot. **(A)** ORR; **(B)** DCR.

**FIGURE 3:**
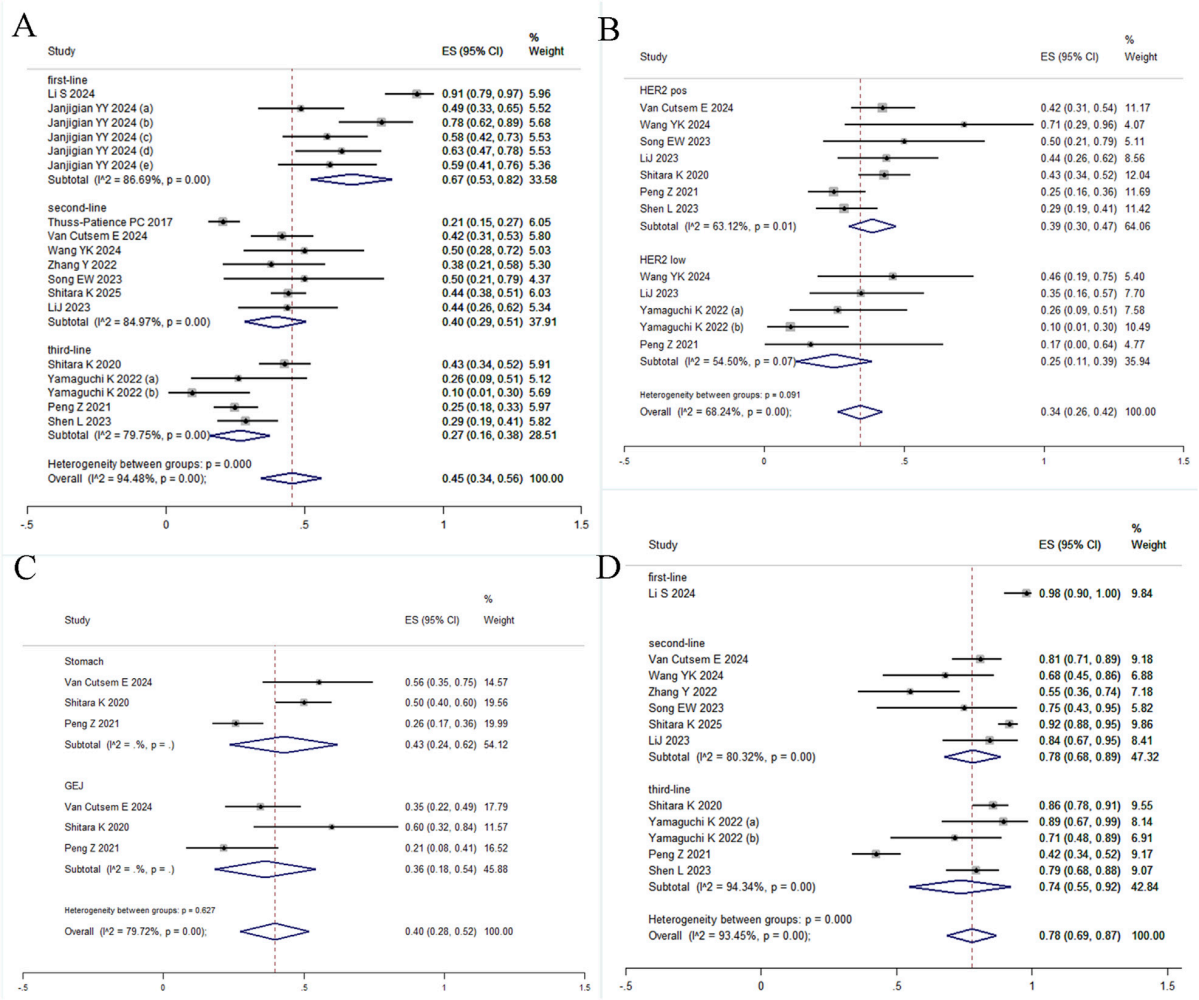
Forest plot of subgroup analyses. **(A)** ORR by treatment line; **(B)** ORR by HER2 status; **(C)** ORR by primary tumor location; **(D)** DCR by treatment line.

All 18 studies reported ORR, with random-effects modeling revealing substantial heterogeneity (*I* ^*2*^ = 94.48%, Q = 308.00) and a pooled estimate of 45% (95% CI: 34%–56%) (Figure 2A). Given significant between-study heterogeneity, meta-regression was conducted and identified study size as a significant effect modifier (*p* = 0.013, adj R^2^ = 31.13%), indicating systematic differences in effect estimates based on trial scale. Neither median age (*p* = 0.073) nor publication year (*p* = 0.320) were significantly associated with outcomes (Supplementary Table S2). To further explore sources of heterogeneity, subgroup analyses stratified by HER2 status, primary tumor location (gastric vs. gastroesophageal junction), and treatment line demonstrated differential efficacy: the pooled ORR was 67% (95% CI: 53%–82%) for first-line therapy, 40% (95% CI: 29%–51%) for second-line therapy, and 27% (95% CI: 16%–38%) for third-line regimens (Figure 3A). In second-line or later therapy, HER2-positive patients exhibited superior ORR *versus* HER2-low subgroups (39%, 95% CI: 30%–47% vs. 25%, 95% CI: 11%–39%) (Figure 3B), while tumor location did not significantly influence outcomes (Figure 3C). Given that 11 of the 18 studies investigated T-DXd, we performed an additional subgroup analysis comparing T-DXd with other ADC agents. The results showed comparable objective response rates between T-DXd and other ADCs (45% [35%–65%] vs. 45% [21%–70%]).

DCR was reported in 12 studies, yielding a pooled estimate of 78% (95% CI: 69%–87%) under random-effects modeling due to substantial heterogeneity (*I* ^*2*^ = 93.45%, Q = 167.81; Figure 2B). Subgroup analyses were restricted to treatment lines given limited trial availability. The pooled DCR was 98% (95% CI: 90%–100%) for first-line therapy, 78% (95% CI: 68%–89%) for second-line therapy, and 74% (95% CI: 55%–92%) for third-line regimens. These findings should be interpreted with caution, as only one study reported outcomes in the first-line setting (Figure 3D). T-DXd demonstrated a significantly higher DCR compared to other ADC agents (85% [79%–91%] vs. 71% [46%–95%]).

### 3.3 OS and PFS

**FIGURE 4:**
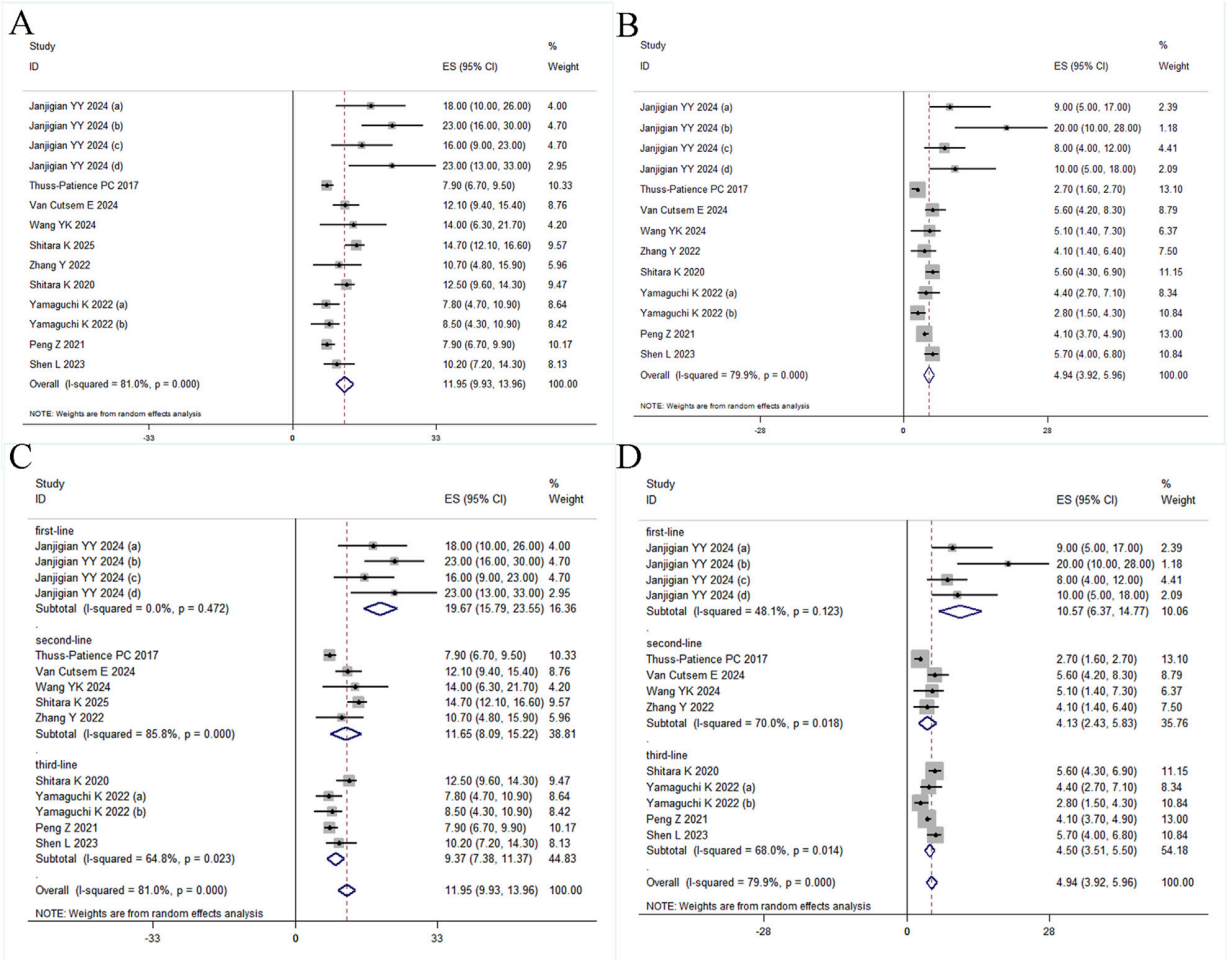
Forest plot. **(A)** OS, **(B)** PFS, **(C)** Subgroup analyses for OS by treatment line, and **(D)** Subgroup analyses for PFS by treatment line.

Fourteen and thirteen studies reported median OS and PFS with 95% CIs, respectively. Pooled analyses yielded median OS of 11.95 months (95% CI: 9.93-13.96) and PFS of 4.94 months (95% CI: 3.92-5.96) (Figures 4A,B), with significant heterogeneity observed for both endpoints (OS: *I* ^*2*^ = 81.0%, Q = 68.47; PFS: *I* ^*2*^ = 79.9%, Q = 59.81). Meta-regression indicated that no covariates significantly predicted heterogeneity in OS. However, study size showed a borderline trend for OS (*p* = 0.067, adj R^2^ = 26.17%). In contrast, for PFS, study size showed a notable association (*p* = 0.058) that accounted for 47.37% of the observed heterogeneity, suggesting that trial scale may substantially influence PFS effect estimates. Neither median age nor publication year were significantly associated with either OS or PFS outcomes (Supplementary Table S2). Subgroup stratification by treatment line (Figures 4C,D) documented distinct survival profiles: first-line (OS 19.67 months (15.79-23.55), PFS 10.57 months (6.37-14.77)), second-line (OS 11.65 months (8.09-15.22), PFS 4.13 months (2.43-5.83)), third-line (OS 9.37 months (7.38-11.37), PFS 4.50 months (3.51-5.50)), with concurrent reduction in heterogeneity magnitude. An additional subgroup analysis comparing T-DXd with other ADC agents was performed. The results showed a median OS of 13.13 months (95% CI, 10.70-15.56) compared to 8.14 months (95% CI, 7.02-9.26), and a median PFS of 5.90 months (95% CI, 4.37-7.43) compared to 3.67 months (95% CI, 2.56-4.78).

### 3.4 Safety

**FIGURE 5:**
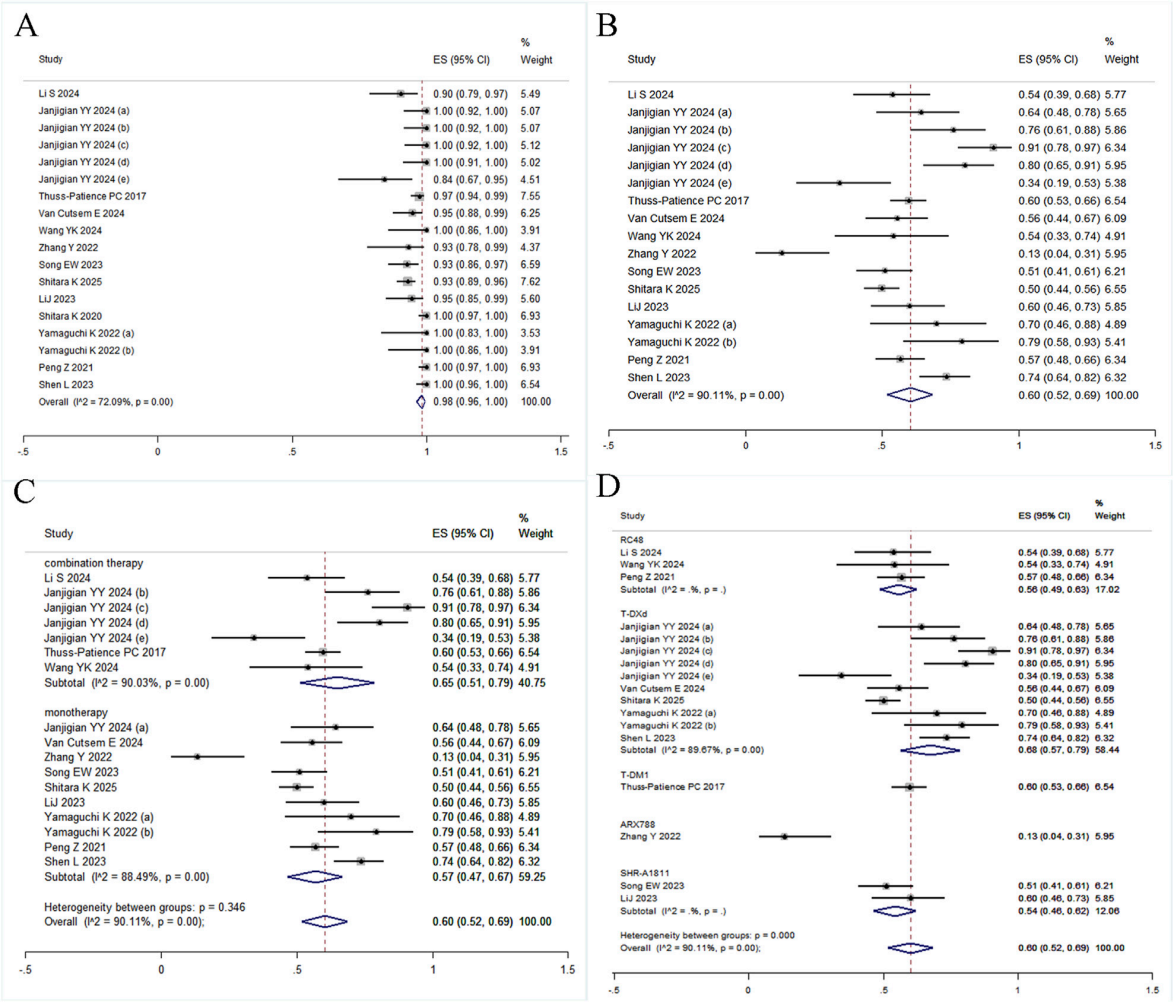
Forest plots of safety. **(A)** any-grade TRAEs, **(B)** grade 3–5 TRAEs, **(C)** Subgroup analysis for grade 3–5 TRAEs by treatment regimens, **(D)** Subgroup analysis for grade 3–5 TRAEs by different ADC agents.

Safety analyses included eighteen studies reporting any-grade TRAEs and seventeen reporting grade 3–5 TRAEs. Pooled incidence rates were 98% (95% CI: 96%–100%) and 60% (95% CI: 52%–69%) respectively, both exhibiting substantial heterogeneity (Figures 5A,B). Subgroup analysis of grade 3–5 events revealed significantly higher incidence with combination therapy *versus* monotherapy (65%, 95% CI: 51%–79% vs. 57%, 95% CI: 47%–67%) (Figure 5C). Comparative assessment of ADC agents showed ARX788 had significantly lower grade 3–5 TRAEs than other drugs (Figure 5D), though this requires validation in larger trials as only one study reported ARX788 outcomes.

### 3.5 Sensitivity analysis and publication bias

Funnel plots for ORR, DCR, OS, and PFS demonstrated general symmetry (Supplementary Figure S2); however, considerable heterogeneity was observed across studies. Begg’s test indicated no significant publication bias (ORR *p* = 0.762, DCR *p* = 0.276, OS *p* = 0.070, PFS *p* = 0.179).

## 4 Discussion

HER2 represents the earliest and best-characterized biomarker in GC, remaining a major research focus for ADC-targeted therapies. Trastuzumab emtansine (T-DM1) received FDA (Food and Drug Administration) approval in 2013 for HER2-positive metastatic breast cancer (Verma et al., 2012). However, the GATSBY trial demonstrated no OS or PFS benefit with T-DM1 *versus* taxanes in HER2-positive advanced GC patients, likely attributed to HER2 heterogeneity within these tumors (Thuss-Patience et al., 2017). Consequently, characterizing heterogeneous HER2 expression emerges as a critical research priority. Compared to T-DM1, T-DXd exhibits potent bystander effects due to enhanced membrane permeability, enabling targeting of GC with low HER2 expression or heterogeneity. RC48, another ADC, received NMPA (National Medical Products Administration) China approval in 2021 and demonstrates promising antitumor activity in GC. Furthermore, investigational ADCs including ARX788 show considerable therapeutic promise.

ADCs enhance the therapeutic index of anticancer treatments by combining the precision of monoclonal antibodies with the potency of cytotoxic agents, thereby reducing off-target toxicity (Staudacher and Brown, 2017; Kalim et al., 2017). An ADC consists of three key elements: an antibody, a linker, and a cytotoxic payload. The antibody, typically a humanized IgG isotype (often IgG1 for its strong effector functions), confers target specificity and must exhibit low immunogenicity, high affinity, and efficient internalization (Khongorzul et al., 2020; Beck et al., 2017). The linker ensures stability in circulation and enables specific payload release at the tumor site; it may be cleavable or non-cleavable, influencing both stability and potential bystander effects (Kalim et al., 2017). The payload, which is highly potent and stable, causes cell death through mechanisms such as microtubule disruption or DNA damage (Li et al., 2016; Puthenveetil et al., 2016).

Following the ToGA trial, trastuzumab combined with chemotherapy became the standard first-line treatment for HER2-positive advanced GC. The ToGA results demonstrated that adding trastuzumab to chemotherapy significantly improved median OS (13.8 months vs. 11.1 months) and median PFS (6.7 months vs. 5.5 months) in patients with HER2-positive metastatic GC, alongside an enhanced ORR (47% vs. 35%; *p* = 0.0017) (Bang et al., 2010). Subsequently, the KEYNOTE-811 study showed that adding pembrolizumab to trastuzumab and chemotherapy further extended median OS (20.0 months vs. 16.8 months) and PFS (10.0 months vs. 8.1 months) compared to trastuzumab and chemotherapy alone. The overall ORR also increased by 12.5% (72.6% vs. 60.1%). However, patients with PD-L1 combined positive score (CPS) < 1 derived no significant benefit in OS, PFS, or ORR from the addition of pembrolizumab (Janjigian et al., 2024b). Pooled analysis of included studies revealed that first-line ADCs for HER2-positive advanced GC achieved integrated efficacy outcomes: ORR 67% (95% CI: 53%–82%), median OS 19.67 months (95% CI: 15.79-23.55), and median PFS 10.57 months (95% CI: 6.37-14.77). Compared to historical controls, these meta-analysis findings indicate superior clinical benefits.

Effective standard second-line anti-HER2 therapies remain limited for patients with advanced HER2-positive GC following progression on first-line anti-HER2 treatment. Results from three phase 3 trials demonstrated that second-line docetaxel or irinotecan improves OS compared with best supportive care in advanced GC (Thuss-Patience et al., 2011; Kang et al., 2012; Ford et al., 2014). A separate phase 3 trial reported comparable OS benefits between irinotecan and paclitaxel (Hironaka et al., 2013). Collectively, these findings have established irinotecan, docetaxel, and paclitaxel as viable second-line chemotherapy options. Ramucirumab, administered either as monotherapy or in combination with paclitaxel, has also demonstrated OS prolongation in the second-line setting for previously treated advanced GC (Wilke et al., 2014; Fuchs et al., 2014). Specifically, the combination of ramucirumab plus paclitaxel significantly extended PFS (4.4 months vs. 2.9 months; *p* < 0.0001) and OS (9.6 months vs. 7.4 months; *p* = 0.0169) compared to placebo, with an ORR of 28%. Results from the present meta-analysis indicate that when used as second-line treatment, the ADC was associated with a pooled median OS of 11.65 months (95% CI: 8.05-15.22), a pooled median PFS of 4.13 months (95% CI: 2.43-5.83), and a pooled ORR of 40% (95% CI: 29%–51%). While PFS showed no improvement, both OS and ORR demonstrated clinically meaningful improvements in indirect comparisons with established regimens. Treatment options for third-line advanced GC remain highly limited. Irinotecan, taxanes, trifluridine/tipiracil, and ICIs (immune checkpoint inhibitors) represent alternative therapeutic approaches (Muro et al., 2019). This meta-analysis demonstrated that for patients receiving the ADC as third-line therapy, the pooled median OS was 9.37 months (95% CI: 7.38-11.37) and the pooled median PFS was 4.50 months (95% CI: 3.51-5.50). The pooled ORR was 27% (95% CI: 16%–38%).

In an ideal scenario, ADCs deliver cytotoxic payloads directly to tumor cells via antibody-mediated targeting, potentially reducing adverse events associated with conventional chemotherapy. However, due to current technological and manufacturing limitations, coupled with variations in antibody specificity, linker stability, and the nature of the cytotoxic payload among different ADC types, significant adverse events may still occur (Wolska-Washer and Robak, 2019). ADC-related toxicities can be categorized based on target antigen involvement: on-target and off-target toxicity. On-target toxicity arises when the ADC specifically binds to and is internalized by antigen-expressing normal tissues, leading to payload release and subsequent cytotoxicity. While the ideal target antigen is highly expressed on tumor cells with minimal or no expression on normal cells, low-level expression on healthy tissues can result in misdelivery of the payload, causing on-target effects. Off-target toxicity refers to damage inflicted by the ADC on organs or cells lacking target antigen expression (Kang and Kim, 2025). Furthermore, adverse events are also intrinsically linked to the payload’s mechanism of action. In HER2-targeting ADCs for GC, common payloads include microtubule inhibitors (e.g., DM1/DM4, MMAE/MMAF) and DNA topoisomerase I inhibitors (e.g., DXd, SN-38).

In this meta-analysis, the pooled incidence rates for any-grade TRAEs and grade 3–5 TRAEs were 98% (95% CI: 96%–100%) and 60% (95% CI: 52%–69%), respectively. The safety profile of ADCs in GC/GEJC generally aligned with established profiles in breast cancer. Subgroup analysis of grade 3–5 TRAEs revealed a higher incidence with combination therapy *versus* monotherapy (65%, 95%CI: 51%–79% vs. 57%, 95%CI:47%–67%). ADC combination regimens-typically incorporating chemotherapy, ICIs, or both-are associated with heightened toxicity. Notably, the observed incidence of grade ≥3 TRAEs in this analysis is comparable to that of established standard regimens in GC/GEJC, such as trastuzumab plus chemotherapy (68% in the ToGA study (Bang et al., 2010)) and pembrolizumab plus trastuzumab and chemotherapy (58% in the Keynote-811 trial (Janjigian et al., 2024b)). Despite the encouraging antitumor activity demonstrated by ADCs in this setting, the considerable incidence of grade 3-5 toxicities cannot be overlooked and necessitates vigilant monitoring, proactive management, and thorough patient counseling to ensure a favorable risk-benefit profile. Comparative assessment of ADC agents indicated that ARX788 demonstrated a notably lower incidence of grade 3–5 TRAEs (13%, 95% CI: 4%–31%) than other evaluated drugs. However, the randomized phase III ACE-Breast-02 trial, comparing ARX788 to lapatinib plus capecitabine in HER2-positive advanced breast cancer, reported similar rates of any-grade and grade ≥3 TRAEs between treatment arms. Specifically, grade ≥3 TRAEs occurred in 41.4% (91/220) of ARX788 recipients (Hu et al., 2025). The incidence of grade 3–5 TRAEs observed with ARX788 in the GC/GEJC setting requires further validation in larger trials.

Several potential limitations warrant consideration. First, our meta-analysis is limited by the predominance of single-arm studies, given the nascent stage of ADC application in advanced GC and the scarcity of large-scale RCTs. This design lacks randomization and a control group, which introduces significant risks of selection bias and confounding. The patients enrolled in these trials are often highly selected based on strict eligibility criteria, such as good performance status and normal organ function, and therefore may not be representative of the broader patient population treated in real-world clinical practice. Consequently, the pooled efficacy outcomes may reflect an optimistic estimate of the treatment’s true effect size, as they could be influenced by a patient cohort with a more favorable prognosis. Furthermore, without a control group, it is challenging to distinguish the treatment effect from the natural disease course or effects of prior therapies. Smaller sample sizes in these studies also increase susceptibility to bias. Thus, the overall findings should be interpreted with caution and are best validated by future large-scale, prospective randomized controlled trials. Second, the paucity of studies within individual treatment lines-exemplified by only two trials in first-line therapy, seven in second-line, and four in third-line settings for ADCs in advanced gastric cancer-necessitated the inclusion of all therapy lines (first-, second-, third-, and later-line) in the pooled analysis. This heterogeneity may introduce confounding factors; thus, subgroup analyses stratified by treatment line were performed to examine potential outcome differences across these strata. Third, the overrepresentation of studies investigating a specific ADC agent, T-DXd, may limit the generalizability of our pooled estimates to other ADC agents. Among these agents, T-DXd represents a preferred therapeutic option where available, given its robust evidence base and superior survival outcomes observed in our analysis. Nevertheless, further studies are warranted to validate the efficacy of other ADC agents and to enable more comprehensive comparative assessments.

## 5 Conclusion

This meta-analysis demonstrates that ADC therapy confers meaningful clinical benefit in patients with advanced GC/GEJC, irrespective of HER2 status (positive or low). These agents maintained a manageable safety profile, which nevertheless requires vigilant monitoring and proactive management. Available evidence indicates that for HER2-positive patients in the first-line setting, conventional standard regimens remain the preferred option. For both HER2-positive and HER2-low populations in the second-line or later settings, ADC therapy emerges as a valuable treatment option.

## Data Availability

The original contributions presented in the study are included in the article/Supplementary Material, further inquiries can be directed to the corresponding author.

## Abbreviations

GC, gastric cancer; GC/GEJC, gastric/gastroesophageal junction adenocarcinoma; HER2, human epidermal growth factor receptor 2; CI, confidence interval; TRAE, treatment-related adverse event; RCT, randomized controlled trial; ASCO, American Society of Clinical Oncology; ESMO, European society of medical oncology; PRISMA, Preferred Reporting Items for Systematic Reviews and Meta-Analyses; OS, overall survival; PFS, progression-free survival; ORR, objective response rate; DCR, disease control rate; IHC, immunohistochemistry; ISH, *in situ* hybridization; ICIs, immune checkpoint inhibitors; EMR, endoscopic mucosal resection; ESD, endoscopic submucosal dissection.
