# Supplementary material for: Efficacy and safety of antibody-drug conjugates for HER2-expressing advanced gastric and gastroesophageal junction adenocarcinoma: a systematic review and meta-analysis
Source: Front Pharmacol. 2025 Sep 17;16:1668511. doi: 10.3389/fphar.2025.1668511 (PMC12484031; doi:10.3389/fphar.2025.1668511)
Supplement: Supplementary file 1 [file Supplementaryfile1.docx]

**Supplementary Table 1**: MINORS score of the included articles

| Criterion | Study | | | | | | | | |
| --- | --- | --- | --- | --- | --- | --- | --- | --- | --- |
|  | Li S 2024 | Janjigian YY 2024 (a) | Janjigian YY 2024 (b) | Janjigian YY 2024 (c) | Janjigian YY 2024 (d) | Janjigian YY 2024 (e) | Thuss-Patience PC 2017 | Van Cutsem E 2024 | Wang YK 2024 |
| 1.A stated aim of the study | 2 | 2 | 2 | 2 | 2 | 2 | 2 | 2 | 2 |
| 2. Inclusion of consecutive patients | 2 | 2 | 2 | 2 | 2 | 2 | 2 | 2 | 2 |
| 3. Prospective collection of data | 2 | 2 | 2 | 2 | 2 | 2 | 2 | 2 | 2 |
| 4. Endpoint appropriate to the study aim | 2 | 2 | 2 | 2 | 2 | 2 | 2 | 2 | 2 |
| 5. Unbiased evaluation of endpoints | 1 | 1 | 1 | 1 | 1 | 1 | 2 | 2 | 1 |
| 6. Follow-up period appropriate to the major endpoint | 1 | 2 | 1 | 1 | 1 | 1 | 2 | 2 | 1 |
| 7. Loss to follow up not exceeding 5% | 2 | 2 | 2 | 2 | 2 | 2 | 2 | 2 | 2 |
| 8. Prospective calculation of the study | 2 | 2 | 2 | 2 | 2 | 2 | 2 | 2 | 2 |
| Size sum | 14 | 15 | 14 | 14 | 14 | 14 | 16 | 16 | 14 |

| Criterion | Study | | | | | | | | |
| --- | --- | --- | --- | --- | --- | --- | --- | --- | --- |
|  | Zhang Y 2022 | Song EW 2023 | LiJ 2023 | Shitara K 2025 | Shitara K 2020 | Yamaguchi K 2022 (a) | Yamaguchi K 2022 (b) | Peng Z 2021 | Shen L 2023 |
| 1.A stated aim of the study | 2 | 2 | 2 | 2 | 2 | 2 | 2 | 2 | 2 |
| 2. Inclusion of consecutive patients | 2 | 2 | 2 | 2 | 2 | 2 | 2 | 2 | 2 |
| 3. Prospective collection of data | 2 | 2 | 2 | 2 | 2 | 2 | 2 | 2 | 2 |
| 4. Endpoint appropriate to the study aim | 2 | 2 | 2 | 2 | 2 | 2 | 2 | 2 | 2 |
| 5. Unbiased evaluation of endpoints | 1 | 1 | 1 | 2 | 2 | 2 | 2 | 2 | 1 |
| 6. Follow-up period appropriate to the major endpoint | 1 | 1 | 1 | 2 | 2 | 1 | 2 | 2 | 2 |
| 7. Loss to follow up not exceeding 5% | 2 | 2 | 2 | 2 | 2 | 2 | 2 | 2 | 2 |
| 8. Prospective calculation of the study | 2 | 2 | 2 | 2 | 2 | 2 | 2 | 2 | 2 |
| Size sum | 14 | 14 | 14 | 16 | 16 | 15 | 16 | 16 | 15 |

**Supplementary Table 2**: Univariable Meta-Regression Analyses for Sources of Heterogeneity in primary outcomes

| primary outcomes | median age | | publication year | | study size | |
| --- | --- | --- | --- | --- | --- | --- |
|  | *p* | adj R^2^ | *p* | adj R^2^ | *p* | adj R^2^ |
| ORR | 0.073 | 16.24% | 0.320 | 0.87% | 0.013 | 31.13% |
| OS | 0.655 | -17.13% | 0.202 | 7.21% | 0.067 | 26.17% |
| PFS | 0.358 | -3.58% | 0.356 | -0.49% | 0.058 | 47.37% |


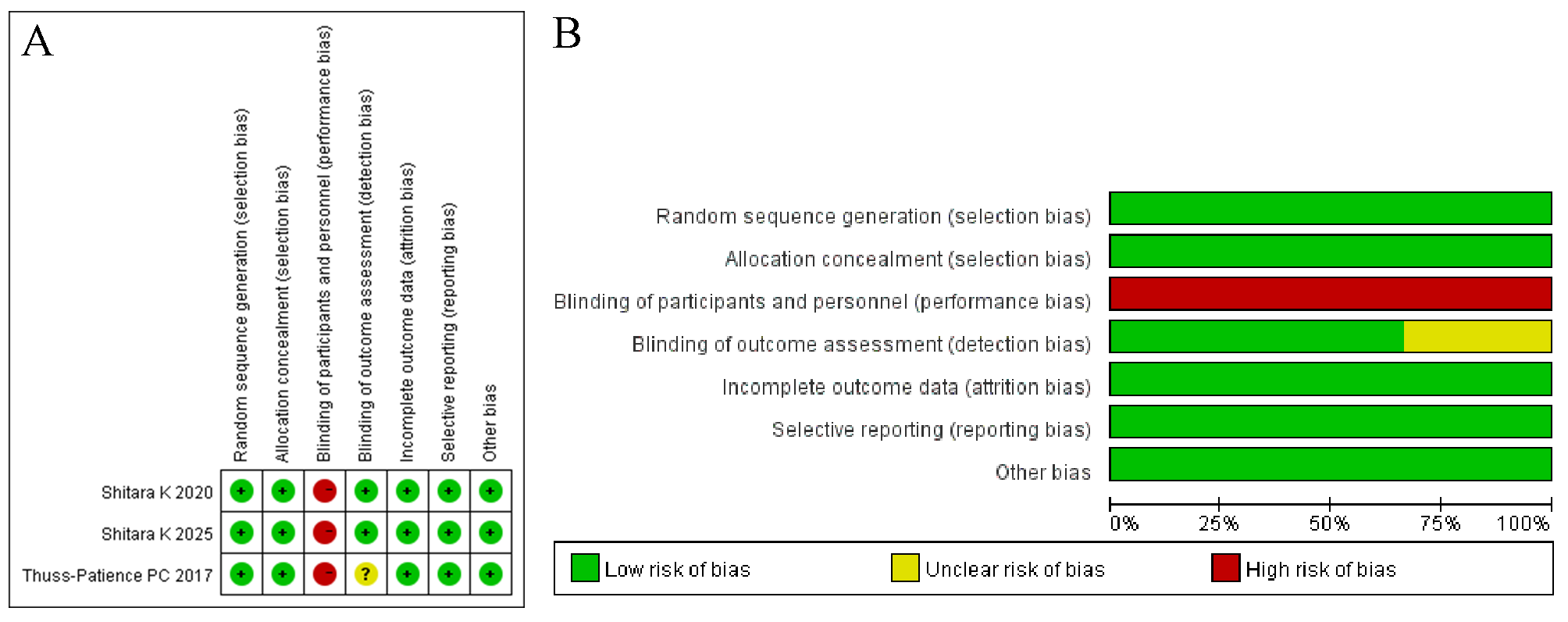


**Supplementary Figure 1**: (A) Risk of bias summary and (B) risk of bias graph.


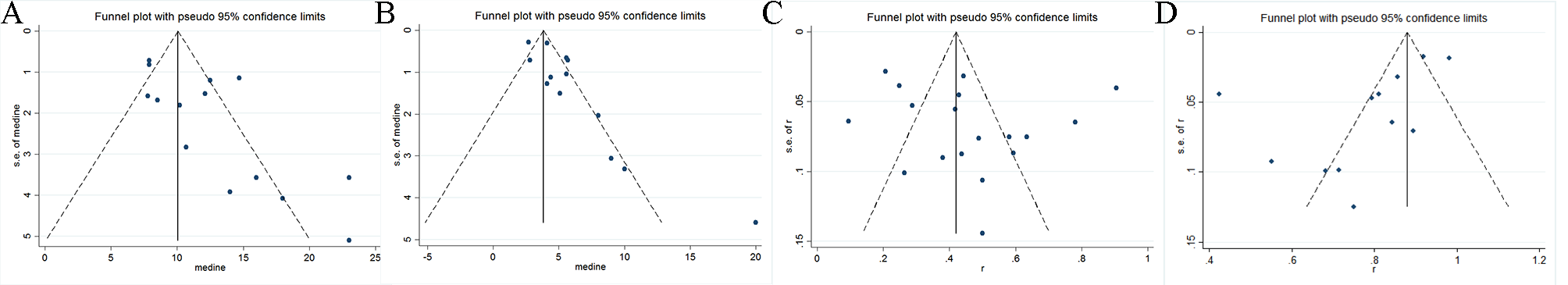


**Supplementary Figure 2**: Funnel plot asymmetry test for publication bias. (A) OS; (B) PFS; (C) ORR; and (D) DCR.
